# Case Report: Gastrointestinal cancer initially presenting as postmenopausal osteoporosis

**DOI:** 10.3389/fonc.2026.1802750

**Published:** 2026-04-29

**Authors:** Jingyuan Li, Tian Liu, Ying Chen, Yang Xu, Lidan Ma, Xiaoyu Cheng, Fei Yan, Ying Gong, Ruixia Sun

**Affiliations:** Department of Endocrinology and Metabolism, The Affiliated Hospital of Qingdao University, Qingdao, China

**Keywords:** bone metastases, colonic adenocarcinoma, gastric adenocarcinoma, postmenopausal osteoporosis, secondary osteoporosis

## Abstract

**Background:**

Gastric cancer usually presents late with nonspecific symptoms such as epigastric discomfort, anorexia, weight loss, nausea, anemia, or gastrointestinal bleeding. Initial presentation with osteoporosis-like manifestations and multiple bone metastases in the absence of gastrointestinal symptoms is rare.

**Case presentation:**

We report a 59-year-old postmenopausal woman who presented with thoracolumbar back pain after routine activity. Bone mineral density assessment showed osteoporosis, but laboratory evaluation revealed markedly increased bone turnover markers, including alkaline phosphatase, β-CTX, and P1NP. Because these findings were disproportionate to typical primary postmenopausal osteoporosis, secondary osteoporosis and other metabolic bone disorders were further investigated. Whole-body bone scintigraphy, SPECT/CT, and PET/CT demonstrated multiple osteoblastic skeletal lesions suggestive of metastatic disease. Gastrointestinal endoscopy subsequently identified two synchronous primary tumors: a poorly differentiated gastric adenocarcinoma with focal signet-ring cell differentiation and a moderately differentiated sigmoid colon adenocarcinoma. Bone biopsy confirmed metastatic adenocarcinoma of gastrointestinal origin, most likely from the stomach. No definite extraosseous visceral metastases were identified on imaging.

**Discussion:**

This case illustrates that disseminated gastric cancer may rarely present without gastrointestinal symptoms and may initially mimic postmenopausal osteoporosis, particularly when bone pain coexists with markedly elevated bone turnover markers. It also highlights the importance of a multidisciplinary diagnostic approach in patients initially suspected of primary osteoporosis but showing atypical biochemical or imaging findings. Clinicians should remain alert to secondary osteoporosis, including occult malignancy, in such settings.

## Introduction

Osteoporosis affects approximately 19.7% of the global population, and primary osteoporosis is the most common form, mainly associated with postmenopausal status and age-related hormonal changes (1). In China, the prevalence of osteoporosis is 19.2% among individuals aged ≥50 years and 32.1% among women (2). Our patient initially presented with osteoporosis without gastrointestinal symptoms. Laboratory tests revealed markedly elevated alkaline phosphatase levels and high bone turnover, findings that mimicked Paget’s disease; however, histopathology confirmed gastric cancer with bone metastases. Although bone metastases are typically associated with breast, lung, and prostate cancers, gastric cancer presenting with bone metastases as the initial manifestation in the absence of gastrointestinal symptoms is rare (3, 4).

## Narrative

The patient was a 59-year-old woman, with no chronic illnesses or long-term medication use, who was admitted due to “thoracolumbar back pain for 13 days, worsening over the last 10 days.” The pain initially developed after a yoga session and was relieved by recumbency, without associated numbness or functional limitation. Ten days before admission, the pain worsened after a jarring taxi ride, resulting in difficulty turning in bed and sitting up. At the initial evaluation in the spine surgery department, magnetic resonance imaging (MRI) of the thoracolumbar spine revealed degenerative changes, including depressions of the T8 and T12 vertebral endplates suggestive of Schmorl’s nodes, along with abnormal vertebral marrow signals; a hematologic disorder was also considered and investigated. Bone mineral density (BMD) assessment confirmed osteoporosis, with a lumbar spine T-score of -3.3, a left femoral neck T-score of -2.4, and a total hip T-score of -2.4. Serum protein electrophoresis, immunoglobulin testing, and κ/λ light chain assays were subsequently performed, and hematologic disease was excluded, after which the patient was admitted to the endocrinology department.

On physical examination, the patient had a body mass index (BMI) of 27.2 kg/m² and was conscious and alert. No kyphosis was observed, the thyroid gland was not enlarged, and there was no chest wall tenderness. Cardiopulmonary auscultation was unremarkable. The abdomen was soft, without tenderness or palpable masses. Spinal percussion tenderness was present, and spinal mobility was restricted. No lower extremity edema was noted. Laboratory evaluation of bone turnover markers (BTMs) showed elevated ALP (562 U/L), β-CTX (1.30 ng/mL), and P1NP (511 ng/mL), together with reduced 25(OH)D (11.00 ng/mL). Sex hormones were consistent with postmenopausal status. Thyroid, adrenal, and pituitary function tests, as well as rheumatologic evaluations, were unremarkable. Among tumor markers, only carcinoembryonic antigen (CEA) was elevated (7.41 ng/mL). More details are in **Table 1**.

**Table 1:** Laboratory test results on admission.

Parameter	Value	Normal range	Units
Albumin	43.4	40-55	g/L
ALT	18	7-40	U/L
AST	20	13-35	U/L
Creatinine	40	41-73	μmol/L
eGFR	109.47	–	mL/min/1.73m^2^
Serum sodium	142.3	137-147	mmol/L
Serum potassium	4.12	3.5-5.3	mmol/L
Serum magnesium	0.94	0.75-1.02	mmol/L
Serum phosphorus	1.35	0.85-1.51	mmol/L
Serum calcium	2.07	2.11-2.52	mmol/L
24h urinary calcium	0.342	0.1-0.3	g/24h
FECa	2.24	–	%
PTH	41.40	15-65	pg/mL
ALP	562	50-135	U/L
25(OH)D	11.00	7.0-53.2	ng/mL
β-CTX	1.30	<1.008	ng/mL
P1NP	511.00	20.25-76.31	ng/mL
CEA	7.41	0-5	ng/mL
AFP	2.39	0-10	ng/mL
CA19-9	5.31	0-37	U/mL

ALT, alanine transaminase; AST, aspartate aminotransferase; FECa, fractional excretion of calcium; PTH, parathyroid hormone; ALP, alkaline phosphatase; P1NP, procollagen-1 intact N-terminal pro-peptide; CEA, carcinoembryonic antigen; AFP, alpha-fetoprotein; CA19-9, carbohydrate antigen.

**Figure 1:**
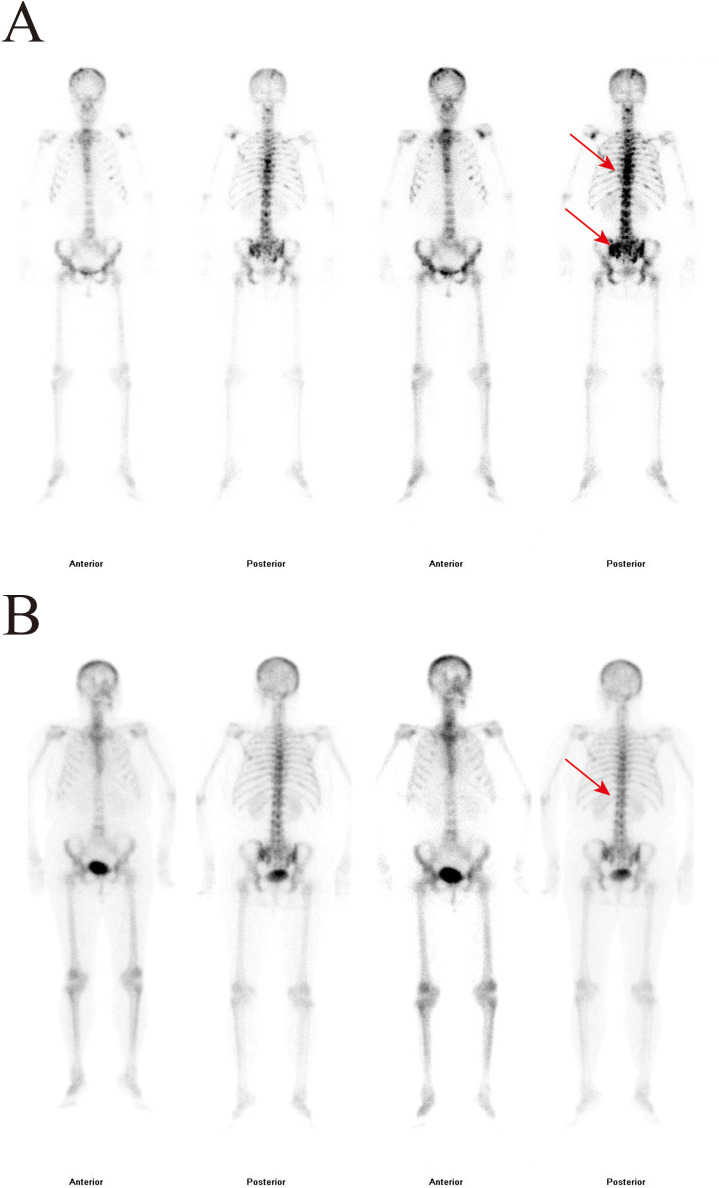
Whole-body bone scintigraphy (WBBS) at baseline and after treatment. **(A)** Baseline WBBS showing multiple foci of increased tracer uptake in the axial skeleton, consistent with multiple osteoblastic bone lesions. **(B)** Follow-up WBBS after treatment showing reduced extent and intensity of abnormal tracer uptake compared with baseline. Red arrows indicate representative sites of abnormal increased tracer uptake, suggesting metastatic osteoblastic bone involvement.

**Figure 2:**
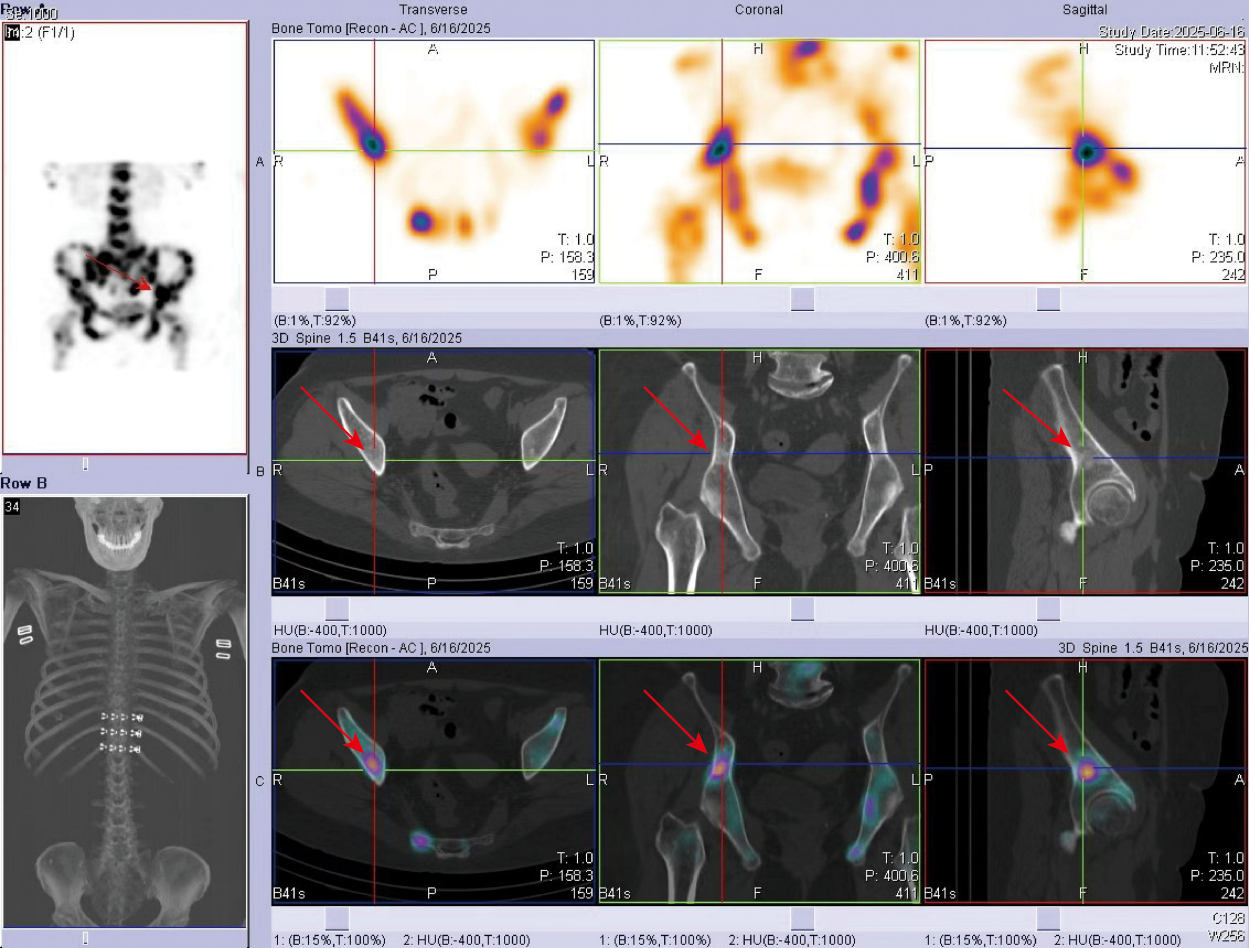
Single-photon emission computed tomography (SPECT/CT) of the pelvis. SPECT/CT demonstrated abnormal radiotracer uptake corresponding to patchy, mottled, and cotton-wool–like hyperdense lesions with marginal osteosclerotic changes in the iliac bone, supporting metastatic bone involvement. Red arrows indicate the representative pelvic lesion identified on axial, coronal, and sagittal images.

**Figure 3:**
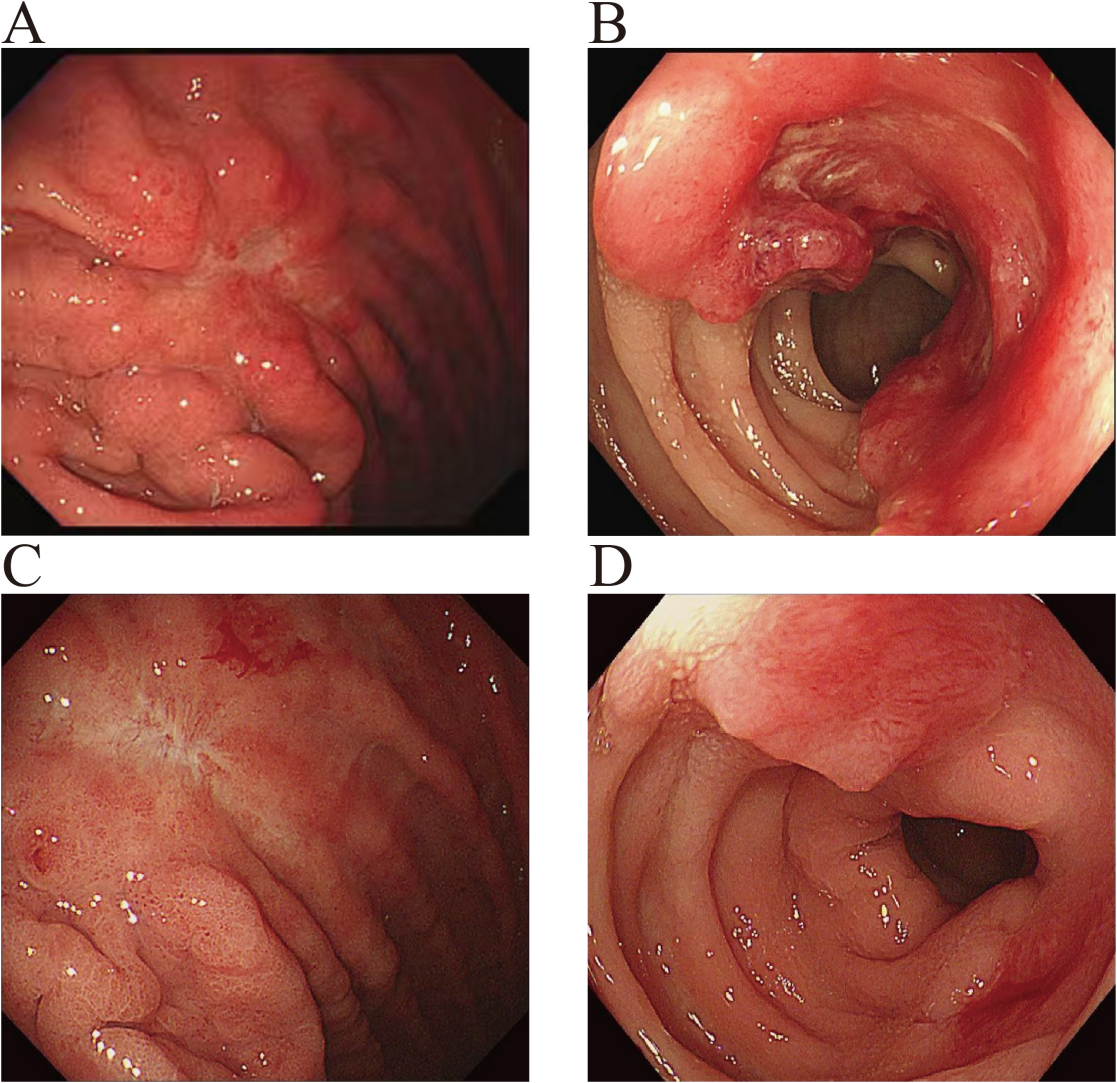
Gastrointestinal endoscopic findings before and after treatment. **(A)** Baseline gastroscopy showing an ulcerative lesion in the gastric body. **(B)** Baseline colonoscopy showing a type 3 lesion in the sigmoid colon. **(C)** Follow-up gastroscopy after treatment showing regression of the gastric body lesion. **(D)** Follow-up colonoscopy after treatment showing regression of the sigmoid colonic lesion.

After admission, the patient received calcium carbonate D3 tablets combined with calcitriol for calcium supplementation, along with intravenous zoledronic acid (5 mg) for osteoporosis. Because primary osteoporosis rarely presents with markedly elevated BTMs, secondary causes of osteoporosis, particularly metabolic bone disorders, needed to be excluded. Whole-body bone scintigraphy (WBBS) (**Figure 1A**) and single-photon emission computed tomography (SPECT/CT) (**Figure 2**) were performed, revealing multiple osteoblastic lesions with abnormal tracer uptake and positron emission tomography–computed tomography (PET/CT) confirmed multiple sites of bone destruction with increased metabolic activity (cervical, thoracic, lumbar-sacral vertebrae, ribs, scapulae, and femora; SUV_max_ = 5.9), suggesting a high probability of bone metastases. Additionally, distal sigmoid colon wall thickening with pronounced metabolic activity (SUV_max_ =30.2) raised concern for colonic malignancy. In addition, PET/CT showed no evidence of distant metastasis involving the liver, lungs, or peritoneum. The patient denied recent weight loss, nausea, heartburn, abdominal pain, or hematochezia. Gastrointestinal endoscopy revealed an ulcerative lesion in the gastric body, pathologically diagnosed as poorly differentiated adenocarcinoma with focal signet-ring cell differentiation, as well as type 3 sigmoid colon cancer, which was identified as moderately differentiated adenocarcinoma (**Figures 3A, B**). Subsequent bone biopsy demonstrated metastatic adenocarcinoma, with immunohistochemistry: CKpan (+), CK7 (+), CK20 (weak +), CDX-2 (+), Villin (+), GATA3 (–), Pax-8 (-), ER (-), TTF-1 (-), p40 (-), CEA (+), consistent with a gastrointestinal origin, most likely gastric. The final diagnoses were secondary osteoporosis (SOP), gastric poorly differentiated adenocarcinoma with bone metastases and sigmoid colon adenocarcinoma. The patient was subsequently transferred to the oncology department and commenced XELOX chemotherapy (oxaliplatin 210 mg on day 1 plus capecitabine 1.5 g twice daily on days 1–4, every 3 weeks) and remains on treatment.

After 6 cycles of XELOX combined with zolbetuximab, together with zoledronic acid for the management of bone metastases-related skeletal involvement, follow-up gastroscopic biopsy showed no residual tumor cells, whereas colonoscopic biopsy continued to demonstrate residual adenocarcinoma (**Figures 3C, D**). WBBS also showed improvement compared with the previous findings (**Figure 1B**), suggesting an overall favorable treatment response. After multidisciplinary team (MDT) discussion, the bone metastases were considered more likely to have originated from the gastric primary lesion. Because the gastric lesion was a poorly differentiated adenocarcinoma with focal signet-ring cell differentiation, suggesting aggressive biological behavior, and because curative gastric surgery would have required total gastrectomy with a substantial adverse impact on quality of life, gastric resection was not pursued, and systemic therapy was continued. As residual disease remained in the colorectal lesion and surgical resection was considered relatively less invasive, laparoscopic-assisted radical resection of the cancer was performed. Intraoperatively, the tumor was located at the rectosigmoid junction, measured approximately 2 × 2 cm, and had not invaded the serosal surface or the pelvic wall. Postoperative histopathology showed ypT2N0M0 disease, with proficient mismatch repair (pMMR) status and HER2 negativity (score 0). The patient recovered well after surgery and subsequently continued maintenance therapy with capecitabine plus zolbetuximab.

## Discussion

Osteoporosis is a systemic bone disorder characterized by reduced bone mass and deterioration of bone microarchitecture, leading to increased skeletal fragility and fracture risk (2). According to its etiology, osteoporosis can be broadly classified as primary or secondary (2). Primary osteoporosis includes postmenopausal osteoporosis (PMO), senile osteoporosis, and idiopathic osteoporosis, whereas secondary osteoporosis results from disrupted bone homeostasis caused by conditions such as endocrine and metabolic disorders, renal insufficiency, and medications (2). PMO typically develops within 5 to 10 years after menopause (5). However, up to 30% of postmenopausal women with osteoporosis may actually have secondary osteoporosis (6). Primary osteoporosis may remain asymptomatic in the early stage, but disease progression can lead to bone pain, kyphosis, or fragility fractures. Dual-energy X-ray absorptiometry (DXA) for the assessment of bone mineral density (BMD) remains the standard diagnostic method. Laboratory findings in primary osteoporosis are usually unremarkable, with serum calcium, phosphate, and alkaline phosphatase levels remaining within the normal range and BTMs being only mildly elevated (2). Markedly elevated BTMs should prompt suspicion of high-turnover secondary osteoporosis. In this case, the patient, who was 10 years postmenopausal, presented with bone pain and low bone mineral density and was initially considered to have PMO. However, the markedly elevated BTMs necessitated further evaluation to exclude secondary osteoporosis and other metabolic bone disorders. A detailed medication history and laboratory evaluation excluded common causes of secondary osteoporosis; however, metabolic bone disorders and malignancy-related bone metastases remained important differential diagnoses.

WBBS has certain advantages in differentiating secondary osteoporosis from other skeletal diseases. Characteristic scintigraphy patterns can be observed in conditions including hyperparathyroidism, Paget’s disease of bone, fibrous dysplasia, osteomalacia, and metastatic bone tumors (2, 7). PET/CT also has diagnostic value in the differential diagnosis of osteoporosis differentiation In this case, the patient exhibited markedly elevated BTMs and multiple sites of increased tracer uptake on WBBS, warranting consideration of Paget’s disease in the differential diagnosis. Paget’s disease of bone, the second most prevalent bone disorder after osteoporosis (8, 9), is a chronic focal skeletal disease that typically involves one or several bones, resulting in enlargement and deformity (10). Its hallmark pathological feature is excessive osteoclastic activity accompanied by compensatory osteoblastic proliferation, which is often associated with markedly elevated BTMs (11). Characteristically, WBBS in Paget’s disease demonstrates markedly increased tracer uptake: homogeneous uptake of the entire vertebra and pedicles (“picture-frame sign” or “Mickey Mouse sign”), diffuse skull uptake (“helmet sign”), and longitudinal extension in long bones with a V-shaped or flame-like pattern (12, 13). However, the scintigraphy findings in this patient did not correspond to the typical imaging features of Paget’s disease.

Bone metastases represent a frequent cause of secondary osteoporosis. Prostate cancer, breast cancer, carcinoid tumors, and small-cell lung carcinoma often give rise to osteoblastic metastases, whereas lymphoma and multiple myeloma typically present with osteolytic lesions, commonly associated with elevated ALP and hypercalcemia (14). In this patient, breast and thyroid examinations, as well as chest CT, showed no abnormalities, and hematologic evaluation excluded these conditions. Bone metastases typically involve the axial skeleton, including the spine, ribs, pelvis, and skull. On WBBS, bone metastases generally appear as focal, irregular areas of tracer uptake with a spotty or patchy distribution and poorly defined margins. The uptake is usually less intense than that seen in Paget’s disease, with overall preservation of bone morphology and no evidence of bony expansion (15). In this case, bone SPECT/CT demonstrated patchy, mottled, and cotton-wool-like hyperdense lesions, together with marginal osteosclerotic changes, findings consistent with osteoblastic bone metastases.

When bone metastases are suspected but the primary tumor remains uncertain, PET/CT combined with bone biopsy can help establish the primary site. In the present case, PET/CT revealed a colonic lesion, and subsequent gastrointestinal endoscopy confirmed synchronous lesions in the stomach and colon. Bone biopsy with immunohistochemistry subsequently confirmed that the bone metastases originated from the stomach. Gastric malignancies typically have an insidious onset, and their clinical manifestations are often nonspecific, including epigastric discomfort, abdominal pain, anorexia, weight loss, nausea, anemia, and gastrointestinal bleeding (16). Due to the absence of characteristic early clinical manifestations, diagnosis is often delayed until the disease has reached an advanced or even metastatic stage (16). Gastric cancer most commonly metastasizes to the liver, lungs, and peritoneum, typically in advanced stages, predominantly through hematogenous and lymphatic dissemination. Bone metastases from gastric cancer are uncommon, and presentation without gastrointestinal symptoms or involvement of other metastatic sites is exceedingly rare (3, 17, 18). Several mechanisms may explain this pattern: 1. Batson’s vertebral venous plexus may serve as a potential metastatic pathway. This valveless venous plexus permits bidirectional blood flow and is widely interconnected with the thoracoabdominal venous system. Under conditions of increased intra-abdominal pressure, such as coughing or defecation, gastric cancer cells may bypass the portal and pulmonary circulation and disseminate directly through the vertebral venous plexus to the spine, pelvis, and other skeletal sites (19). 2. Bone marrow homing may also contribute. Chemokines secreted by the bone matrix, such as SDF-1α, recruit CXCR4-expressing gastric cancer cells to the bone marrow. Through adhesion molecules, tumor cells attach to and transmigrate across the vascular endothelium into the bone marrow niche, where they may be supported by osteoclast-derived growth factors including IGF-1 and PDGF, ultimately establishing metastatic lesions (14, 20, 21).

Postmenopausal osteoporosis is the most common cause of primary osteoporosis in women. However, when bone turnover markers are markedly elevated, secondary osteoporosis should be carefully excluded. Bone scintigraphy and PET/CT play important roles in distinguishing primary from secondary osteoporosis, and bone biopsy may be useful when the diagnosis remains uncertain.

## Conclusion

Although postmenopausal osteoporosis is common in clinical practice, up to 30% of postmenopausal women with osteoporosis may have secondary osteoporosis. This case involved a postmenopausal woman with secondary osteoporosis and multiple vertebral lesions who was ultimately diagnosed with bone metastases from gastric adenocarcinoma. It underscores the importance of distinguishing secondary osteoporosis, particularly when abnormal bone turnover markers cannot be explained by primary osteoporosis alone. Early multidisciplinary evaluation is crucial for timely diagnosis and prompt initiation of systemic treatment.

## Patient perspective

The patient was relieved that the cause of her back pain was identified and expressed gratitude for the multidisciplinary care she received. Although the diagnosis was difficult to accept, she felt reassured by timely treatment and consented to publication of her case to help raise awareness of secondary osteoporosis.

## Data Availability

The raw data supporting the conclusions of this article will be made available by the authors, without undue reservation.
